# Application of the novel peroral choledochoscope in hepatolithiasis after liver transplantation

**DOI:** 10.1055/a-2767-0903

**Published:** 2026-03-02

**Authors:** Ximin Lin, Wen Cheng, Xiaokui Qiu, Jiawen Sun, ShaoBo Chen, Huan Peng, Zhongming Dai

**Affiliations:** 1 673618 Department of Gastroenterology, Shenzhen Guangming District Peopleʼs Hospital, Shenzhen, China

Hepatolithiasis (HL) is a refractory benign biliary disease 1 . HL frequently recurs and is difficult to cure completely 2 , rendering its surgical therapy challenging. It also is a rare and complex complication after liver transplantation 3 . Herein, we report the use of a novel biliary choledochoscope (EyeMax Choledochoscope System Digital Controller; Micro-Tech, Nanjing, China) for the treatment of HL after liver transplantation.

**Fig. 1:**
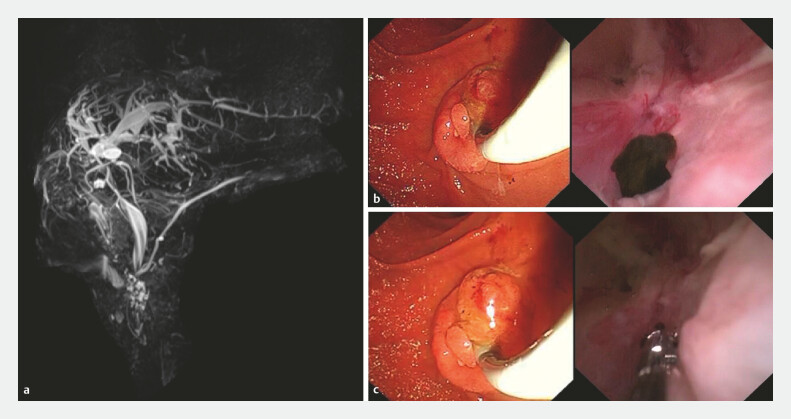
**a** In a 58-year-old man with a history of intermittent right upper abdominal pain after orthotopic liver transplantation, magnetic resonance cholangiopancreatography revealed a focal stenosis of the hilar bile duct and dilation of the left intrahepatic bile ducts. **b** The peroral choledochoscope was advanced into the left intrahepatic bile duct, revealing a dark brown stone. **c** Under direct visualization, the stone was grasped and pulled into the duodenum.

A 58-year-old man was admitted with a history of recurrent right upper quadrant abdominal pain for over 3 years. Twenty years prior, he had undergone orthotopic liver transplantation for decompensated hepatitis B-related cirrhosis. Physical examination revealed tenderness in the right upper quadrant, with no other remarkable findings. Routine laboratory testing results revealed the following levels: a total bilirubin of 34.6 µmol/L and a direct bilirubin of 18.6 µmol/L. Magnetic resonance cholangiopancreatography ( Fig. 1 **a** ) demonstrated a focal stenosis of the hilar bile duct and dilation of the left intrahepatic bile ducts. With the patient’s consent, an exploratory procedure using the novel peroral choledochoscope was recommended. During the procedure, cannulation of the distal common bile duct was achieved with a 0.025-inch guidewire-assisted sphincterotome (Boston Scientific, USA). Subsequently, the peroral choledochoscope was advanced through the working channel of the duodenoscope into the left intrahepatic bile duct, revealing a dark brown stone ( Fig. 1 **b** ). The biliary biopsy forceps (Micro-Tech, Nanjing, China; Fig. 1 **c** ) was introduced through the working channel of the peroral choledochoscope. Under direct visualization, the stone was grasped and pulled into the duodenum. Finally, repeated exploration of the bile ducts confirmed no residual stones ( Video 1 ). The patient recovered well and was discharged 5 days later.

Hepatolithiasis removal after liver transplantation with the eyeMax choledochoscope.Video 1

Compared to ERCP, the eyeMax pancreatobiliary imaging system provides superior image clarity 4 5 . It compensates for the shortcomings of ERCP, avoids the trauma caused by surgical procedures, and provides new ideas for the treatment of HL after liver transplantation.

Endoscopy_UCTN_Code_TTT_1AR_2AG
